# Impaired osteogenesis in Menkes disease-derived induced pluripotent stem cells

**DOI:** 10.1186/s13287-015-0147-5

**Published:** 2015-09-07

**Authors:** Dongkyu Kim, Jieun Choi, Kyu-Min Han, Beom Hee Lee, Jin-Ho Choi, Han-Wook Yoo, Yong-Mahn Han

**Affiliations:** Department of Biological Science, Korea Advanced Institute of Science Technology (KAIST), Daejeon, 305-701 Republic of Korea; Department of Pediatrics, Asan Medical Center Children’s Hospital, University of Ulsan College of Medicine, Seoul, South Korea

## Abstract

**Introduction:**

Bone abnormalities, one of the primary manifestations of Menkes disease (MD), include a weakened bone matrix and low mineral density. However, the molecular and cellular mechanisms underlying these bone defects are poorly understood.

**Methods:**

We present in vitro modeling for impaired osteogenesis in MD using human induced pluripotent stem cells (iPSCs) with a mutated *ATP7A* gene. MD-iPSC lines were generated from two patients harboring different mutations.

**Results:**

The MD-iPSCs showed a remarkable retardation in CD105 expression with morphological anomalies during development to mesenchymal stem cells (MSCs) compared with wild-type (WT)-iPSCs. Interestingly, although prolonged culture enhanced CD105 expression, mature MD-MSCs presented with low alkaline phosphatase activity, reduced calcium deposition in the extracellular matrix, and downregulated osteoblast-specific genes during osteoblast differentiation in vitro. Knockdown of *ATP7A* also impaired osteogenesis in WT-MSCs. Lysyl oxidase activity was also decreased in MD-MSCs during osteoblast differentiation.

**Conclusions:**

Our findings indicate that *ATP7A* dysfunction contributes to retardation in MSC development and impairs osteogenesis in MD.

**Electronic supplementary material:**

The online version of this article (doi:10.1186/s13287-015-0147-5) contains supplementary material, which is available to authorized users.

## Introduction

Menkes disease (MD) is a copper metabolism disorder that is caused by a loss-of-function of a major copper transporter, ATP7A [1, 2]. The *ATP7A* gene is located on the long arm of X chromosome and encodes a P-type ATPase, which plays crucial roles in cellular copper metabolism by controlling copper export and intracellular copper trafficking [3, 4]. Although its primary physiological function is copper absorption in the small intestine, ATP7A is also implicated in intracellular copper delivery to copper-dependent enzymes [5, 6]. A variety of copper-dependent enzymes become nonfunctional due to a lack of ATP7A activity in MD patients, which can lead to multisystemic clinical symptoms [6, 7].

Clinical manifestations of MD patients include progressive neurodegeneration, connective tissue defects, sparse and kinky hairs, vascular defects, and many others. Connective tissue defects comprise tortuous vessels, skeletal change, loose skin, laxity of joints, and so forth [7–9]. Similarly, MD mouse models present with fragmentation of the internal elastic lamina, defective synthesis of bone collagen, reduced skin tensile strength, and weak blood vessels [10–12]. Among the various symptoms, bone abnormalities are a typical phenotype in MD patients [13–15]. Bone abnormalities in Menkes patients include osteoporosis, metaphyseal spurs, diaphyseal fractures, and wormian occipital bones [15–19]. Bone defects have also been shown in occipital horn syndrome, a mild phenotype of ATP7A-deficient disease [20]. Defective phenotypes in bone formation are frequently used as a diagnostic test in the early stage along with a blood test to measure low serum copper levels [16]. However, in vitro model systems that investigate how ATP7A mutations result in abnormal bone formation in MD have not been reported. Here, we attempt to model MD pathogenesis at the cellular level using induced pluripotent stem cells (iPSCs) in vitro. Human iPSCs, which have the capability to differentiate into various cell types and to proliferate indefinitely, are useful cell sources for studying the pathogenesis of human diseases [21, 22].

In this study, MD-iPSCs were differentiated into osteoblasts (OBs) to investigate the effect of ATP7A dysfunction on bone formation. Intriguingly, MD-iPSCs showed delayed mesenchymal stem cell (MSC) maturation compared with wild-type (WT)-iPSCs. Subsequently, MD-MSCs showed impaired osteogenesis in terms of alkaline phosphatase (ALP) activity, calcium mineralization, and transcription of osteogenic genes. Copper chelation in WT-MSCs resembled defective phenotypes shown in MD-MSCs. Our results demonstrate that dysfunction of copper utilization in MD gives rise to delayed MSC development and impaired OB differentiation.

## Materials and methods

### Retrovirus production

For retrovirus packaging, retroviral vectors encoding OCT4, SOX2, KLF4, cMYC (Addgene, Cambridge, MA, USA) were co-transfected with VSV-G vector (Takara Bio, Mountain View, CA, USA) in GP2 293 cells. Transfectants were further incubated in Dulbecco’s modified Eagle’s medium (DMEM; Welgene, Seoul, Korea) supplemented with 10 % fetal bovine serum (FBS; Invitrogen, Carlsbad, CA, USA) and 1 % penicillin-streptomycin (Invitrogen) at 37 °C, 5 % CO_2_ in air. The medium was changed 8 h after transfection. Then, supernatants were harvested 48 and 72 h after incubation. Supernatants harvested from four dishes (10 cm in diameter) per factor were ultracentrifuged at 90,000 × *g* for 90 min at 4 °C. The viral pellet was dissolved in 2 ml of the medium and kept at −70 °C before use.

### Generation and maintenance of MD-iPSCs

To generate MD-iPSCs, patient fibroblasts were infected with four retroviruses and then plated onto mitomycin C-treated (MMC; A.G. Scientific, San Diego, CA, USA) MEF feeder layers at a density of 10^3^ cells/cm^2^. This study using patient fibroblasts was approved by the Institutional Review Board of Asan Medical Center, and written informed consent was obtained from their parents. Infected cells were cultured in human embryonic stem cell (ESC) medium at 37 °C, 5 % CO_2_ in air for 2 to 3 weeks. The human ESC medium consists of DMEM/F12 (Invitrogen) supplemented with 20 % Knockout SR (Invitrogen), 1 % nonessential amino acids (Invitrogen), 1 % penicillin-streptomycin, 0.1 mM β-mercaptoethanol (Sigma, St. Louis, MO, USA), and 10 ng/ml fibroblast growth factor (FGF)2 (R&D systems, Minneapolis, MN, USA). Respective human ESC-like colonies were mechanically transferred onto new MMC-treated MEF feeders and subcultured for stabilization for 10 to 20 passages. iPSC characteristics were analyzed by the expression of human ESC markers, karyotypes, methylation states on promoters of human ESC marker genes, and teratoma formation. WT-iPSCs derived from foreskin fibroblasts [23] were used as a control group (Additional file 1: Figure S1).

### Real-time quantitative PCR

Total mRNA was extracted from iPSCs and differentiated cells using easy-Blue™ (Intron Biotechnology, Seongnam, Korea). Briefly, approximately 1 × 10^5^ cells were washed in phosphate-buffered saline (PBS) and treated with 1 ml easy-Blue™ solution. After mixing with 200 μl chloroform, cell lysates were centrifuged and the upper layer of the supernatants was harvested to isolate RNA. Then, RNA was precipitated and rehydrated for cDNA synthesis. A total of 1 μg RNA was annealed with oligo(dT), and cDNA was synthesized using M-MLV Reverse Transcriptase (Enzynomics, Daejeon, Korea). The real-time polymerase chain reaction (RT-PCR) was performed using the following cycle conditions: 95 °C denaturation, 60 °C annealing, and 72 °C elongation. The cycle numbers for each reaction varied between 30 and 40. Red safe (Intron Biotechnology) was used for visualization of PCR products in gel electrophoresis. For quantitative comparison, the relative expression level was measured by CFX-Connect real-time system (Bio-Rad, Hercules, CA, USA). The relative expression level of each gene was analyzed using a comparative threshold cycle method, and the transcription level of *GAPDH* was used for normalization. The primers used in this study are listed in Additional file 2 (Tables S1 and S2).

### Bisulfite sequencing

Genomic DNA was isolated from cell samples using a G-DEX Genomic DNA Extraction Kit (Intron Biotechnology). Briefly, 2 × 10^6^ cells were lysed in cell lysis buffer (300 μl) at room temperature for 5 min and then incubated at 37 °C for 30 min in the presence of RNase A. After the addition of PPT Buffer (100 μl), cell lysates were centrifuged at 16,000 × *g* for 5 min, and the supernatant was harvested. Isopropanol (300 μl) was added to the supernatant. After centrifugation at 16,000 × *g* for 1 min, the DNA pellet was dissolved in distilled water (DW). Bisulfite treatment was performed using a Zymo EZ DNA methylation kit (Zymo Research, Irvine, CA, USA) according to manufacturer’s instructions. Briefly, 1 μg of genomic DNA was denatured at 95 °C for 10 min, and CT-conversion was performed by addition of the CT Conversion Reagent. CT-converted DNA was desulfonated in M-Desulfonation Buffer, washed with M-Wash Buffer, and dissolved in 20 μl of DW. Bisulfite-treated genomic DNA was amplified by PCR, individually cloned into a pGEM®-T vector (Promega, Madison, WI, USA), and sequenced using an ABI 3730XL DNA Analyzer (Applied Biosystems, Foster City, CA, USA). Methylation quantification was performed using the QUMA program (Riken, Kobe, Japan). The primers used in bisulfate sequencing are listed in Additional file 2 (Table S3).

### Immunostaining

The cells were fixed with 4 % formaldehyde for 30 min, washed twice in PBST (PBS containing 0.1 % Tween 20), and permeabilized in PBS containing 0.1 % Triton X-100 (Sigma) for 20 min. After blocking with 2 % bovine serum albumin (BSA; Sigma) for 1 h, the cells were treated with each primary antibody and incubated at 4 °C overnight. The primary antibodies used in this study were as follows: OCT4 (Santa Cruz Biotechnology, Santa Cruz, CA, USA); SOX2 (Cell Signaling, Danvers, MA, USA); NANOG (R&D Systems); SSEA4 (Abcam, Cambridge, England); TRA-1-60 (Millipore, Billerica, MA, USA); TRA-1-81 (Millipore); NESTIN (Millipore); α-SMA (R&D Systems); and GATA4 (Santa Cruz Biotechnology). The cells were washed several times in PBST and incubated with secondary antibodies (Alexa Fluor 488 or 594; Invitrogen) for 1 h. Then, the cells were washed several times in PBST and counter-stained with 4′-6-diamidino-2-phenylindole (DAPI; Sigma) during the washing step. After washing with PBST, fluorescence images were observed on a Zeiss LSM 510 confocal microscope equipped with argon and helium–neon lasers (Carl Zeiss, Germany).

### Teratoma formation of MD-iPSCs

Animal care and experimental procedures were performed under the approval of the Animal Care Committees of KAIST. MD-iPSCs (1 × 10^7^ cells) were collected by scraping, mixed with Matrigel (BD Biosciences, Franklin Lakes, NJ, USA), and subcutaneously injected into the dorso-lateral area of CAnN.Cg-Foxn1 nu/CrljOri mice (Orient, Seongnam, Korea). Approximately 2 months after injection, the tumor tissues were dissected and embedded in paraffin wax. Tissue sections were placed on slide glasses. Hematoxylin and eosin (H&E; Sigma) staining was performed to observe various cell types and tissues.

### Differentiation of human iPSCs into MSCs

Differentiation of human iPSCs into MSCs was performed as previously described [24, 25]. Briefly, human iPSC colonies were mechanically dissected and transferred to low-adhesion petri dishes (SPL Lifesciences, Pocheon, Korea). Dissected human iPSCs spontaneously aggregated to form embryoid bodies (EBs) in EB medium at 37 °C with 5 % CO_2_ for 1 day. The EB medium consists of DMEM/F12 supplemented with 10 % Knockout SR, 1 % nonessential amino acids, 1 % penicillin-streptomycin, and 0.1 mM β-mercaptoethanol. EBs were further cultured in the EB medium containing 10 μM SB431542 (Abcam) at 37 °C with 5 % CO_2_ for 10 days, and then attached to fibronectin-coated dishes (BD Biosciences). The attached cells were further cultured in DMEM/F12 supplemented with 1 μM SB431542, 1 % ITS Liquid media supplement (Sigma), 1 % B27 supplement (Invitrogen), and 1 % CD lipid concentrate (Invitrogen) for 4 days. Then, the cells were cultured in α-minimum essential medium (α-MEM; Invitrogen) containing 10 % FBS for 20 days for MSC induction.

### FACS analysis

Cells were dissociated by treatment with trypsin-EDTA (0.25 %; Invitrogen) for 5 min followed by the addition of fresh culture medium containing FBS for enzyme inactivation. After centrifugation at 300 × *g* for 5 min, the pellets were resuspended in FACS buffer (PBS containing 2 % FBS) and filtered through a cell strainer with a 40-μm pore size (SPL Lifesciences). Dissociated cells were incubated with specific FACS antibodies against CD44, CD73, CD90, CD105, and respective isotype controls (Biolegend, San Diego, CA, USA) at 4 °C for 30 min. After washing with FACS buffer, samples were analyzed using a FACSCalibur flow cytometer (BD Biosciences). The positive population for each antibody was evaluated with FlowJo software (Tree Star, Ashland, OR, USA). Gating strategy for this analysis is summarized in Additional file 3 (Figure S2).

### Western blotting

Cells were lysed with Pro-Prep protein extraction solution (Intron Biotechnology) on ice for 1 h. After centrifugation at 16,000 × *g* for 30 min, the supernatant was harvested. The concentration of protein lysates was determined by Bradford protein assay (Bio-Rad). Proteins were loaded onto an SDS-PAGE gel (Elpis Biotech, Daejeon, Korea) and then transferred to a nitrocellulose membrane (Whatman, Maidstone, England). The membrane was blocked with TBST (0.1 % Tween in TBS) containing 4 % skim milk, washed in TBST, and treated with the appropriate primary antibodies. The primary antibodies used in this study were as follows: SMAD2 (Cell Signaling); p-SMAD2 (Cell Signaling); ACTIN (Santa Cruz Biotechnology); ATP7A (Hycult Biotech, Uden, Netherlands); SMAD1 (Cell Signaling); and p-SMAD1 (Cell Signaling). After washing in TBST, the membrane was incubated with an horseradish peroxidase (HRP)-conjugated secondary antibody (Thermo Fisher Scientific, Waltham, MA, USA) at room temperature for 1 h. The membrane was developed using the ECL system (Thermo Fischer Scientific), and images were captured by LAS-3000 (Fuji Film, Tokyo, Japan).

### MSC proliferation and apoptosis

Cells were seeded on a gelatin-coated dish at a density of 2 × 10^3^ cells/cm^2^ and cultured in α-MEM containing 10 % FBS for 9 days. The number of cells was calculated daily using a hemocytometer (Marienfeld, Lauda-Königshofen, Germany). To examine cell viability, cells were first plated on a gelatin-coated dish at a density of 2 × 10^4^ cells/cm^2^ and then cultured for 1 day. After the addition of the CCK-8 reagents (Dojindo, Kumamoto, Japan), the cells were incubated at 37 °C for 30 min, and absorbance was measured at 450 nm. To detect apoptosis, cells were resuspended in 1X annexin V binding buffer (eBioscience, San Diego, CA, USA) and incubated with annexin V-FITC (eBioscience) and propidium iodide (PI; Sigma) for 15 min in the dark. After FACS analysis, annexin-positive cells were counted and graphed. For cell cycle analysis, cells were fixed in cold 70 % ethanol for 1 h and then treated with RNase A (Sigma) for 30 min. After treatment with PI for 15 min, the distribution of cells in the cell cycle was analyzed on a FACSCalibur.

### MSC differentiation into OBs and chondrocytes

For osteogenesis, MSCs were seeded on to gelatin-coated dishes at a density of 2 × 10^4^ cells/cm^2^ and cultured in α-MEM containing 10 % FBS for 1 day. Then, MSCs were cultured in StemPro® Osteogenesis Differentiation Medium (Invitrogen) at 37 °C with 5 % CO_2_ for 21 days. Medium was changed every 3–4 days. Differentiated cells were analyzed by ALP activity, alizarin red S staining, and Von Kossa staining. For chondrogenesis, MSCs were concentrated in α-MEM containing 10 % FBS at a density of 1 × 10^7^ cells/ml. A 10 μl droplet was placed into noncoated wells of a 96-well plate (SPL Lifesciences) for 1 h, and 100 μl StemPro® Chondrogenesis Differentiation Medium (Invitrogen) was added to each well. The next day, the MSC spheroids that formed were further cultured in the same medium for 2–3 weeks. The medium was changed every 3–4 days. The Alcian blue staining method was utilized to confirm chondrogenic differentiation.

### ALP assay

Fixative solution and ALP staining solution are required for the ALP assay. Fixative solution is a mixture of 25 ml citrate solution (Sigma), 65 ml acetone (Junsei Chemical, Tokyo, Japan) and 8 ml 37 % formaldehyde. To create the ALP staining solution, 1 ml sodium nitrite solution (Sigma) was mixed with 1 ml FBV solution (Sigma). After incubation at room temperature for 2 min, 45 ml DW and 1 ml naphthol As-BI alkaline solution (Sigma) were added to the mixture. For the ALP assay, the cells were fixed in fixative for 30 s and then incubated in ALP staining solution for 20 min in the dark.

### Alizarin red S, Von Kossa, and Alcian blue staining

For alizarin red S staining, the cells were fixed with 10 % formalin for 20 min and then incubated with alizarin red S staining solution (Millipore) for 20 min. For Von Kossa staining, cells were fixed with 10 % formalin for 20 min and then exposed to ultraviolet light in 5 % silver nitrate (American Master Tech, Lodi, CA, USA) for 1 h. After washing with DW, the cells were incubated in 5 % sodium thiosulfate (American Master Tech) at room temperature for 3 min and then observed under an inverted microscope (Olympus, Tokyo, Japan). For Alcian blue staining, chondrogenic spheroids were fixed with 10 % formalin for 30 min and embedded in 2 % agarose (LPS solution, Seoul, Korea) in PBS. Sections of chondrogenic spheroids were treated with 3 % acetic acid (Millipore) for 3 min and then incubated in Alcian blue staining solution (American Master Tech) for 30 min. The stains were detected using an inverted microscope.

### Transfection of ATP7A-siRNA into WT-MSCs

Predesigned siRNAs (Bioneer, Daejeon, Korea) targeting *ATP7A* were transfected into WT- MSCs using Lipofectamine® RNAiMAX (Invitrogen). Briefly, a total of 5 pmol siRNA was diluted in 50 μl Opti-MEM® medum (Invitrogen) and mixed with 3 μl Lipofectamine® RNAiMAX diluted in 50 μl Opti-MEM® medum. After 5 min incubation at room temperature, siRNA-lipid complexes were added to the culture medium.

### Lysyl oxidase activity

To measure lysyl oxidase (LOX) activity, supernatants were obtained 7 days after the induction of MSCs to OBs. The LOX assay reaction solution is a mixture of 20 μl Amplite™ HRP substrate stock solution (Abcam), 20 μl HRP (50 U/ml, Abcam), and 5 ml assay buffer (Abcam). A total of 50 μl supernatant and 50 μl LOX assay reaction solution were added to 96 wells. After incubation at 37 °C for 20 min in the dark, fluorescence was measured at Ex/Em = 540/590 using a microreader (Tecan, Maennedorf, Switzerland).

### Matrix collagen assay

To measure collagen deposition in the extracellular matrix (ECM), human iPSC-derived OBs were fixed in 200 μl Kahle fixative solution (Chondrex, Redmond, WA, USA) in four-well dishes for 10 min. After washing with DW, the cells were incubated in 200 μl dye solution (Chondrex) at room temperature for 30 min. After removal of the dye solution, 500 μl dye extraction buffer (Chondrex) was added to elute the bound dye solution. The OD values of the eluted dye solution were measured at 540 nm and 605 nm using a microreader.

### Measurement of intracellular copper concentration

WT-MSCs and MD-MSCs were detached with trypsin-EDTA and digested in a mixture of 2 ml 65 % HNO_3_ (J.T. Baker®, PA, USA), and 7 ml deionized water. Prepared samples were further digested in the Microwave Digestion System (Milestone Inc., CT, USA) for 4 h at 150 °C. Then, copper concentration was analyzed by inductively coupled plasma (ICP) mass spectrometry (Agilent Technologies, CA, USA).

### Statistical analysis

The statistical significance of the real-time RT-PCR data and other assays was evaluated by Student’s *t*-test, and *p* < 0.05 was considered significant.

## Results

### Generation of iPSCs from MD patient fibroblasts

Dermal fibroblasts were obtained from two different patients who each had a mutation in the *ATP7A* gene [26]. The 2-year-old Patient 1 (Menkes disease case 1, MD1) had an intronic mutation (c.4005 + 5G > A) that causes a splicing error on exon 20. The newborn Patient 2 (Menkes disease case 2, MD2) had a large genomic deletion (c.121-930_2626 + 488del) encompassing the exon 3–12 region (Table 1). The mutated region of patient MD1 is in the ATP-binding domain, which modulates the catalytic activity, and the deleted regions of MD2 include five copper-binding domains, four transmembrane regions, and fragments of a phosphatase domain that disturb a large portion of ATP7A (Additional file 4: Figure S3A). The two patients showed typical symptoms of MD, including severe neurodegeneration and intensive connective tissue abnormality (Table 1).

**Table 1:** Genetic information of Menkes patients

Identification number	MD Case1 (MD1)	MD case2 (MD2)
Gender	Male	Male
Age at diagnosis	4 months	36 days
Genotype	c.4005 + 5G > A of ATP7A (Splice site mutation)	c.121-930_2626 + 488del of ATP7A (Large deletion)
Protein	Exon 20 deletion	Exon 3–12 deletion
Manifestations	Lethargy	Diffuse cerebral dysfunction
	Seizure	Developmental delay
	Hypotonia	Hypotonia
	Hypsarrhythmic pattern on EEG	Elongated tortuos intracranial vessels
	Elongated tortuos intracranial vessels	Brittle hair & loose skin
	Brittle hair & loose skin	
Copper (68–168 μg/dl)	17	8
Initial ceruloplasmin (13.1–42.8 mg/dl)	4.6	<3
Current outcomes	Death (4.7 years)	Bed ridden (2.6 years)

**Fig. 1:**
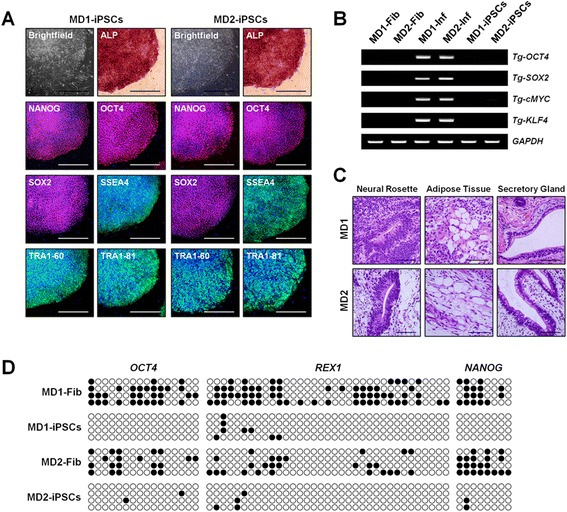
Generation of MD-iPSCs. **a** Expression of pluripotent markers in MD-iPSCs. MD1- and MD2-iPSCs had normal morphologies and expressed pluripotent markers. Scale bars = 500 μm. **b** Transcriptional expression of transgenes such as *OCT4*, *SOX2*, *cMYC*, and *KLF4* in MD-Fib, MD-inf, and MD-iPSCs. Transcription of the transgenes was detected only in infected MD1 and MD2 fibroblasts. **c** Teratoma formation of MD-iPSCs in immunodeficient mice. H&E staining was performed to detect diverse cell types and tissues (neural rosette, ectoderm; adipose tissue, mesoderm; and secretory gland, endoderm). Scale bars = 100 μm. **d** Epigenetic reprogramming in MD-iPSCs. Promoters of pluripotent genes were highly demethylated in MD1- and MD2-iPSCs compared with fibroblasts. Each circle represents the methylation status of single CpG dinucleotides: empty circle, unmethylated; filled circle, methylated. *ALP* alkaline phosphatase, *iPSC* induced pluripotent stem cell, *Inf* infected fibroblasts, *Fib* normal fibroblasts, *MD1/2* Menkes disease patient 1/2, *Tg* transgene

MD-iPSCs were generated from dermal fibroblasts of patients MD1 and MD2 by ectopic expression of OCT4, SOX2, cMYC, and KLF4. A MD1-iPSC clone, a MD2-iPSC clone, and WT-iPSCs were used in this study. MD1- and MD2-iPSCs had a typical morphology with tightly packed clusters and sharp boundaries and expressed pluripotency-associated marker genes (Fig. 1a and Additional file 4: Figure S3B). Exogenous genes were silenced after iPSC generation (Fig. 1b). MD1- and MD2-iPSCs differentiated into various cell types of the three germ layers in vitro (Additional file 4: Figure S3C) and formed teratomas after subcutaneous injection into nude mice (Fig. 1c). Furthermore, methylation of CpG dinucleotides in the promoter of *OCT4*, *NANOG*, *REX1* genes were highly demethylated in MD1- and MD2-iPSCs compared with each patient’s fibroblasts (Fig. 1d), indicating successful epigenetic reprogramming. MD1-iPSCs had a normal karyotype, and MD2-iPSCs showed a polymorphic variant (pericentric inversion of chromosome 9) that was the same as the karyotype of the MD2 patient (Additional file 4: Figure S3D). Mutations of the *ATP7A* gene were confirmed again at the genomic and transcriptional levels in MD1- and MD2-iPSCs (Additional file 4: Figure S3E and S3F).

### Differentiation of MD-iPSCs into MSCs

**Fig. 2:**
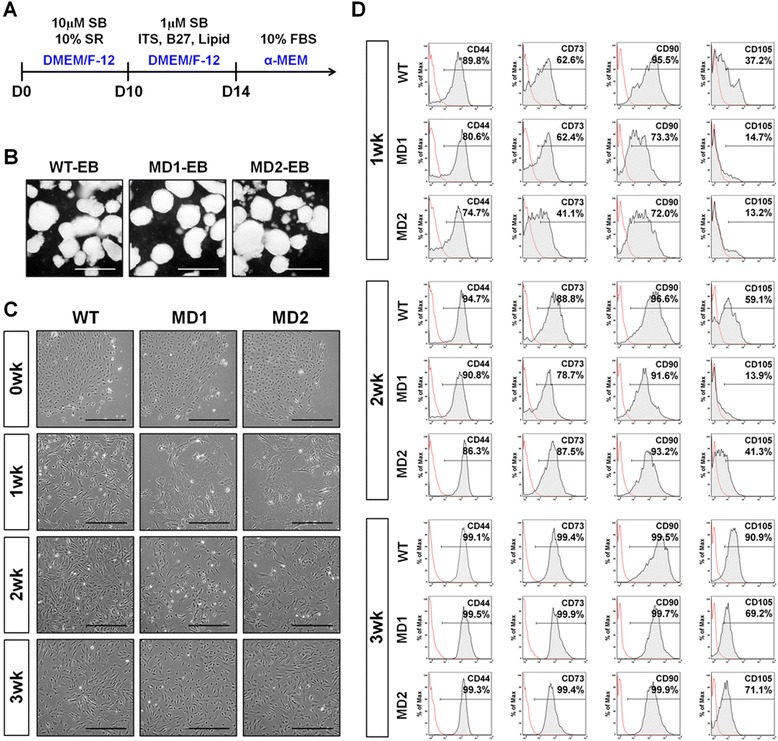
Differentiation of MD-iPSCs into MSCs. **a** Schematic protocol for differentiation of MD-iPSCs into MSCs. **b** Formation of embryoid bodies (EBs) from MD-iPSCs. MD1- and MD2-iPSCs formed EBs with typical morphologies. Scale bars = 500 μm. **c** Representative images of iPSC-derived cells at 0, 1, 2, and 3 weeks after α-MEM induction. Retardation of MSC differentiation was observed both in the MD1- and MD2-iPSC groups compared with the WT-iPSC group. Scale bars = 500 μm. **d** Expression of MSC markers in MD-MSCs. MD1- and MD2-MSCs showed lower expression of CD105 compared with WT-MSCs. Gating strategy for this analysis is summarized in Additional file 3 (Figure S2). *α-MEM* α-minimum essential medium, *D* day of differentiation, *DMEM* Dulbecco’s modified Eagle’s medium, *FBS* fetal bovine serum, *ITS* ITS liquid media supplement, *MD1/2* Menkes disease patient 1/2, *SB* SB431542, *SR* Knockout Serum Replacement, *wk* weeks, *WT* wild type

**Fig. 3:**
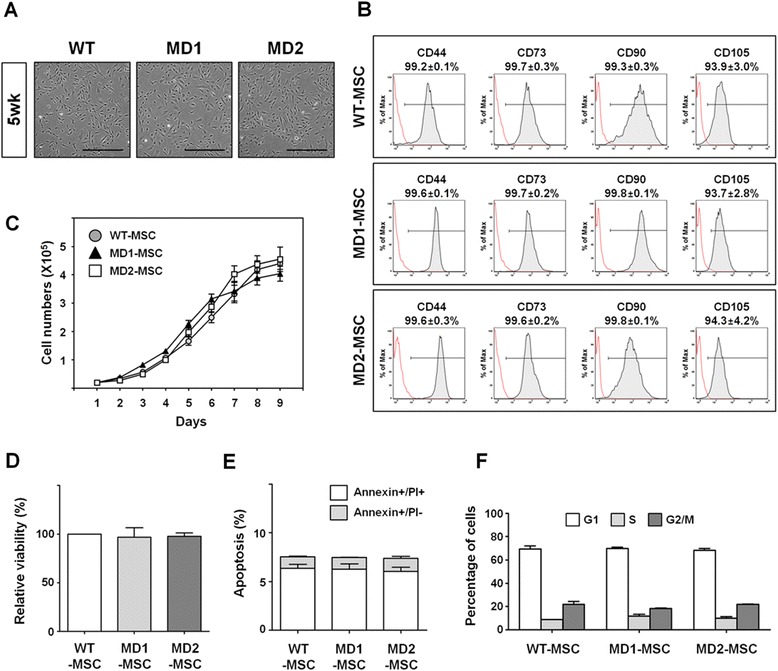
Maturation of MD-MSCs by extended culture. **a** Bright-field microscopy images of MD-MSCs. MD1- and MD2-MSCs were matured by long-term culture for 5 weeks. Scale bars = 500 μm. **b** Expression of MSC surface antigen markers. After long-term culture, CD105-positive cells were increased in MD1- and MD2-MSCs. Gating strategy for this analysis is summarized in Additional file 3 (Figure S2). **c** Growth curves of WT- and MD-MSCs. Each symbol indicates the number of cells at each day of culture. The data are presented as the mean ± SE (n = 4). **d** Viability of WT- and MD-MSCs. Absorbance data of MD-MSCs obtained from a viability assay (see the Materials and Methods section) are expressed as relative to the WT-MSCs. The data are presented as the mean ± SE (n = 3). **e** Apoptosis of WT- and MD-MSCs. The percentage of early apoptotic cells (annexin^+^ and PI^−^) and late apoptotic cells (annexin^+^ and PI^+^) of WT- and MD-MSCs are depicted on a graph. The data are presented as the mean ± SE (n = 2). **f** Cell cycle analysis of WT- and MD-MSCs. Cell cycle distributions of WT- and MD-MSCs were determined by FACS analysis. The percentage of cells in each phase of the cell cycle (G1, S, and G2/M) was quantified and depicted as a graph. The data are presented as the mean ± SE (n = 2). *MD1/2* Menkes disease patient 1/2, *MSC* mesenchymal stem cell, *PI* propidium iodide, *wk* weeks, *WT* wild type

MSCs were differentiated from MD-iPSCs using an EB-based method (Fig. 2a). In this method, EBs were treated with an inhibitor of transforming growth factor-beta signaling, SB431542 (SB), to enhance differentiation into cardiac mesoderm and neuro-ectoderm lineages. Treatment with SB efficiently blocked SMAD2 phosphorylation in all WT, MD1 and MD2 EBs (Additional file 5: Figure S4A). SB-treated EBs were morphologically normal in the three groups (Fig. 2b), and showed upregulated expression of a cardiac mesodermal gene, *cTNT*, and a neuro-ectodermal gene, *NEUROD1*, compared to undifferentiated cells (Additional file 5: Figure S4B). After attachment of SB-treated EBs to fibronectin-coated dishes, development of mesenchymal cells appeared to be retarded in MD-iPSCs (MD1- and MD2-iPSCs) compared with that of WT-iPSCs. Mesenchymal morphology could be observed at 1 week after α-MEM induction in the WT-iPSC group, whereas mesenchymal morphology was observed at 2 weeks in the MD1- and MD2-iPSC groups (Fig. 2c). Differences in mesenchymal development between the WT- and MD-iPSC groups were also apparent after FACS analysis (Fig. 2d). CD105 expression in the MD1- and MD2-iPSC groups was relatively low by 3 weeks during mesenchymal development compared with the WT-iPSC group. The MD1- and MD2-iPSC groups also showed a slight reduction in CD90 expression after 1 week of α-MEM induction, but no difference was detected in the expression of other MSC markers, such as CD44 and CD73, between the WT- and MD-iPSC groups. These results demonstrate that the induction of MD-EBs towards the mesenchyme may be delayed in the early stage. Intriguingly, however, MD1- and MD2-MSCs achieved the normal MSC morphology and cell density of WT-MSCs after a long-term culture of 5 weeks (Fig. 3a). Furthermore, the expression level of CD105 in MD-MSCs was similar to that of WT-MSCs (Fig. 3b), indicating the complete maturation of MSCs. In addition, mature MD-MSCs had normal cellular functions, including cell growth (Fig. 3c), viability (Fig. 3d), apoptosis (Fig. 3e) and cell cycle (Fig. 3f) compared with WT-MSCs. Thus, ATP7A mutations did not influence fundamental cellular functions in MSCs. Genetic mutations of the *ATP7A* gene were confirmed again in the MD1- and MD2-MSCs (Additional file 6: Figure S5A and S5B, respectively). The ATP7A protein was not detected in either the MD1- or MD2-MSCs (Additional file 6: Figure S5C), and MD-MSCs exhibited higher levels of intracellular copper than WT-MSCs (Additional file 2: Table S4).

### Impaired osteogenesis of MD-MSCs

**Fig. 4:**
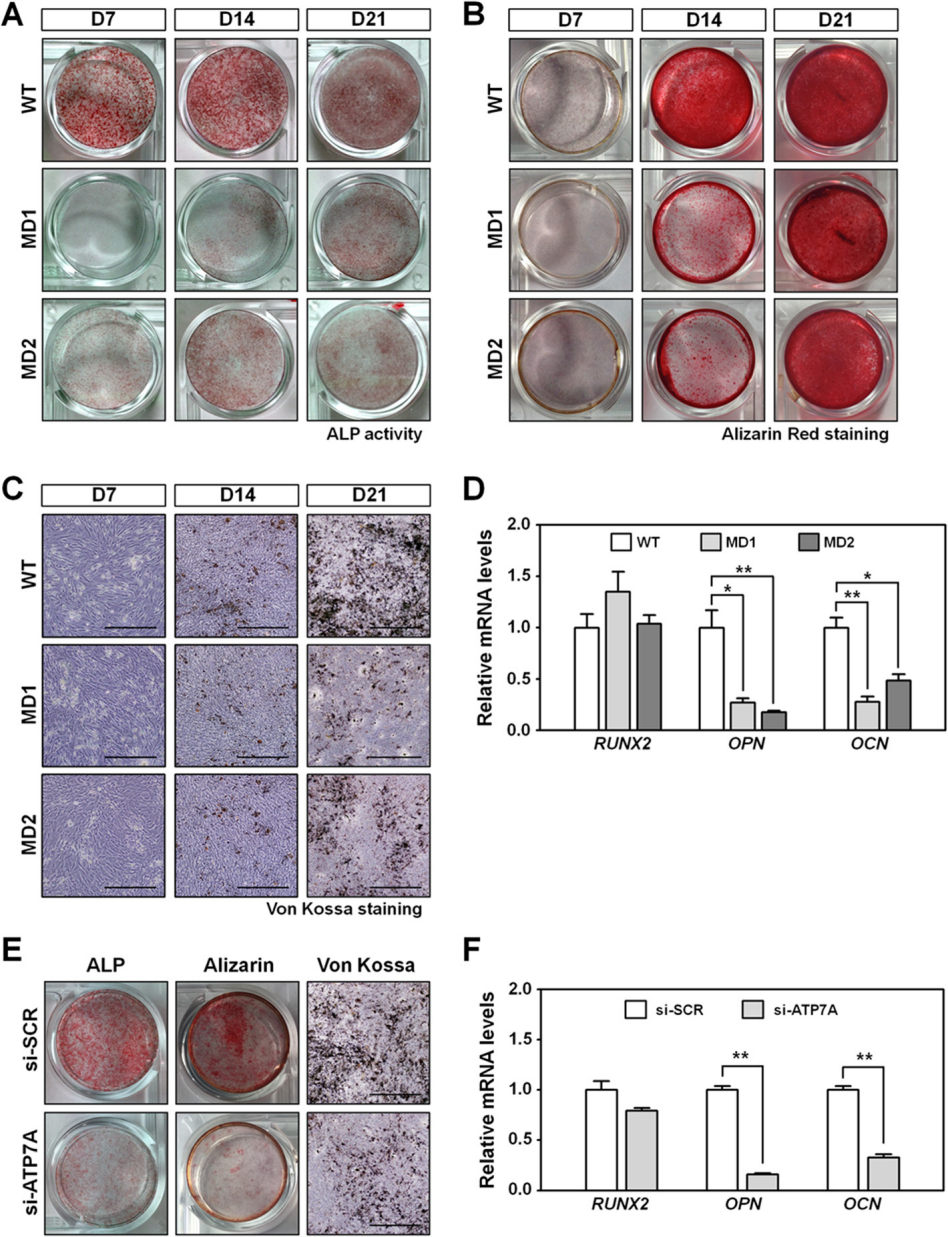
Impaired osteogenesis in MD-MSCs. **a** Representative images of ALP activity in WT- and MD-MSCs during OB differentiation. ALP activity was observed as red granules. D, days after osteogenesis induction. **b** Representative images of alizarin red S staining in WT- and MD-MSCs during OB differentiation. Alizarin red S staining presented as red granules. **c** Representative images of Von Kossa staining in WT- and MD-MSCs during OB differentiation. Von Kossa staining was observed as black dots. Scale bars = 500 μm. **d** Relative expression of osteogenic genes *RUNX2*, *OPN*, and *OCN* in MD-MSCs during osteogenesis. The data are presented as the mean ± SE (n = 3). **e, f** Effects of *ATP7A* knock-down on osteogenesis in WT-MSCs. **e** Representative images of ALP activity, alizarin red S staining, and Von Kossa staining after transfection of siRNAs targeting *ATP7A* gene. Scramble siRNA (si-SCR) was also transfected in WT-MSCs as a control. **f** Relative expression of *RUNX2*, *OPN*, and *OCN* after *ATP7A* knockdown. The data are represented as the mean ± SE (n = 3). *p < 0.05, **p < 0.01. *ALP* alkaline phosphatase, *D* day of differentiation, *MD1/2* Menkes disease patient 1/2, *WT* wild type

To test the effect of *ATP7A* mutations on osteogenesis during bone formation, MD-MSCs were differentiated into OBs. To monitor OB differentiation, an ALP assay, alizarin red S staining, and Von Kossa staining were performed. In WT-MSCs, ALP activity was clearly observed at 7 days and reached the highest level 14 days after OB induction (Fig. 4a, upper panel). Intriguingly, ALP activity was barely detected at 7 days and was very weak even at 21 days in the MD1-MSCs during OB differentiation (Fig. 4a, middle panel). MD2-MSCs also showed low ALP activity in the process of OB differentiation (Fig. 4a, bottom panel). Thus, we found that MD-MSCs had aberrant ALP activity during OB differentiation. ALP activity is very important for calcium crystallization or mineralization during bone formation [27]. Therefore, we speculated that low ALP activity might lead to abnormal calcium deposition in MD-MSCs. As expected, MD1- and MD2-MSCs showed lower levels of calcium deposition during OB differentiation compared with WT-MSCs (Fig. 4b and c). These results imply that reduced ALP activity accounts for insufficient calcium crystallization or mineralization. Relative expression of the matrix-related genes *OPN* and *OCN* was significantly downregulated, whereas the expression of the osteogenic transcription factor *RUNX2* was similar in MD-OBs compared with WT-OBs (Fig. 4d). To test whether ATP7A is associated with osteogenesis, a knockdown experiment using siRNA targeting *ATP7A* was carried out in WT-MSCs. In a preliminary experiment, siRNA use efficiently downregulated *ATP7A* transcript (Additional file 7: Figure S6). Knockdown of *ATP7A* in WT-MSCs also showed impaired osteogenesis with downregulation of osteogenic genes (Fig. 4e and f). Thus, knockdown of ATP7A in WT-MSCs recapitulated osteogenic impairments of MD-MSCs. Our results indicate that ATP7A plays an important role in bone formation.

Previous findings have shown that bone morphogenetic protein 2 (BMP2) induces OB differentiation of MSCs [28, 29]. We therefore examined whether impaired osteogenesis in MD-MSCs is caused by insufficient activation of the BMP2 signaling pathway. No differences were detected in the activity of p-SMAD1 between the WT- and MD-MSCs (Additional file 8: Figure S7A). In addition, chondrogenesis appeared normal in MD-MSCs (Additional file 8: Figure S7B). These results suggest that decrements of ALP activity and mineralization adversely affect osteogenesis in MD-MSCs. Next, activity of LOX, a copper-dependent enzyme, was measured to test whether it is associated with osteogenesis. The MD1- and MD2-OBs showed lower LOX activity than WT-OBs (Additional file 9: Figure S8A), but the amount of matrix collagen was not different between WT- and MD-OBs (Additional file 9: Figure S8B). Therefore, it is conceivable that impaired mineralization is not due to reduced deposition of collagen in MD-MSCs. Taken together, we suggest that the ATP7A mutation causes decreased ALP activity and mineralization, eventually resulting in impaired osteogenesis in MD.

## Discussion

Here, we provide novel insight into the impaired osteogenesis in MD using iPSCs. ATP7A, which is a major copper transporter, plays important roles in copper absorption and delivery of copper to the human body [6, 8]. Copper is one of the essential trace elements in normal development, and its homeostasis should be tightly regulated [30]. Disability of copper utilization in MD patients who have a defective *ATP7A* gene causes severe multisystemic phenotypes such as connective tissue abnormalities. In this study, MD-iPSCs with *ATP7A* mutations showed retardation of MSC development (Fig. 2d), although MD-MSCs matured after extended culturing in vitro (Fig. 3). Subsequently, several osteogenic defects, including decreased ALP activity and weak calcium mineralization, were observed in MD-MSCs and ATP7A-knockdown WT-MSCs during OB differentiation (Fig. 4).

ALP activity appears to be associated with calcium deposition during osteogenesis. During osteogenesis, ALP produces inorganic phosphate (Pi) from pyrophosphate (PPi), and controls the balance between Pi and PPi levels in the ECM [31, 32]. Pi is further crystallized with calcium and accelerates calcium mineralization in the ECM. Dysfunction of the *ALP* gene causes a genetic disorder called hypophosphatasia, which is characterized by abnormal bone formation [27]. Here, low activity of ALP resulted in decreased calcium mineralization in MD-MSCs during OB differentiation as shown by alizarin red S and Von Kossa staining (Fig. 4).

LOX mediates cross-linking of collagen and elastin in the ECM, which enhance tensile strength and structural integrity of connective tissues [3]. It has been postulated that decreased LOX activity accounts for the impaired connective tissue in MD patients [33, 34]. Reduced activity of LOX is also implicated in abnormal vasculopathy such as the vascular tortuosity and peripheral aneurysms in MD and its allelic variant, occipital horn syndrome (OHS) [35]. Aberrant internal elastic lamina structure is observed in the MD patient and the Menkes mouse model [36]. Cultured fibroblasts of MD and OHS show abnormalities in the expression of connective tissue genes [37]. Furthermore, bladder diverticula, inguinal hernia, skin laxity, hyperelasticity, and occipital exostosis are caused by reduced LOX activity [38].

In this study, LOX activity was decreased in MD-MSCs during OB differentiation. Nonetheless, there were no changes in collagen deposition in this study. These results raise the possibility that another role of LOX might be involvement in the aberrant OB differentiation in MD-MSCs. In fact, it has been reported that LOX activity is involved in many biological functions other than collagen cross-linking, such as metastasis, tumor cell growth, cell migration and motility, angiogenesis, cell signaling, and transcription [39–42]. Thus, the identification of a new role for LOX in MD-MSCs during OB differentiation would be very interesting.

Taken together, these data show that utilization of intracellular copper is unavailable in MD cells due to dysfunctional ATP7A. This study provides additional insight into the pathophysiology of bone defects caused by the *ATP7A* mutation in MD.

## Conclusions

Here we described the important role of ATP7A and copper during osteogenesis using MD-derived iPSCs. During OB differentiation, several osteogenic impairments such as low ALP activity, reduced calcium mineralization and decreased expression of osteogenic marker genes were observed. Knockdown of ATP7A in WT-MSCs recapitulated the impaired osteogenesis observed in MD-MSCs. Our results provide new insight into the important role of ATP7A in bone formation.

## Additional files

Additional file 1: Figure S1. Characterization of WT-iPSCs. (A) Expression of pluripotent markers in WT-iPSCs. (B) Teratoma formation of MD-iPSCs in immunodeficient mice. (C) Epigenetic reprogramming in WT-iPSCs. (TIFF 2486 kb) Additional file 2: Table S1. Primers used in RT-PCR analysis. **Table S2** Primers used in ATP7A genotyping of MD-derived cells. **Table S3** Primers used in methylation analysis. **Table S4** Copper concentration in WT- and MD-MSCs. (DOCX 19 kb) Additional file 3: Figure S2. Detailed gating strategy for each MSC surface antigens. Gating strategy for CD44 surface antigen shown as a representative. Live cells (gate A) were gated based on forward scatter and side scatter. Staining with isotype control (PE-conjugated) was used to exclude CD44-negative events. This gate was then applied to samples stained with anti-CD44 antibody (PE-conjugated) to identify CD44 positive events. In merged image, red line indicates isotype control and black tinted area indicates CD44 positive events. (TIFF 1455 kb) Additional file 4: Figure S3. Characterization of MD-iPSCs. (A) Schematic defective regions of ATP7A in MD1 and MD2 patients. The functional domains of ATP7A are depicted. ATP7A has six copper-binding domains, one phosphatase domain, one phosphorylation domain, one ATP-binding domain, and eight transmembrane domains. The defective regions of each patient are marked as boxes. (B) Transcriptional expression of pluripotent genes in MD-fibroblasts and MD-iPSCs. *GAPDH* was used as a control. (C) In vitro differentiation of MD-iPSCs. Immunostaining of NESTIN (ectoderm, red), α-SMA (mesoderm, green), and GATA4 (endoderm, green) was performed 7 days after spontaneous EB differentiation. DAPI showed nuclear counterstaining (blue). Scale bar = 500 μm. (D) Karyotypes of MD1- and MD2-iPSCs. (E) Genetic mutations in MD1-fibroblasts and MD1-iPSCs. A single-base substitution was confirmed in MD1-fibroblasts and MD1-iPSCs (Figure E-a). The resultant PCR products were different in MD1-fibroblasts and MD1-iPSCs (Figure E-b). The size difference (450 bp in WT and 246 bp in MD1) generated by exon skipping was analyzed by gel electrophoresis. (F) Mutations in MD2-fibroblasts and MD2-iPSCs. Strategy for duplex PCR was explained as an illustration (Figure F-a). Size differences (532 bp in WT and 224 bp in MD2) generated by a large genomic deletion between WT- and MD2-fibroblasts were detected (Figure F-b). The primers used in this study are listed in Additional file 1 (Table S2). (TIFF 4321 kb) Additional file 5: Figure S4. Characterization of EBs during MSC differentiation. (A) SMAD2 phosphorylation in WT- and MD-EBs during MSC differentiation. SB treatment suppressed p-SMAD2 in WT- and MD-EBs. (B) Relative expression of neuro-ectoderm and cardiac mesoderm marker genes (*NEUROD1* and *cTNT,* respectively) in WT- and MD-EBs. The data are presented as the mean ± SE (*n* = 3); **p* < 0.05, ***p* < 0.01. (TIFF 2052 kb) Additional file 6: Figure S5. Confirmation of MD-MSCs. (A) Genetic mutation of the *ATP7A* gene in MD1-MSCs. A single-base substitution was confirmed again in MD1-MSCs. (B) Genetic mutation of the *ATP7A* gene in MD2-MSCs. Size differences in the PCR product were observed in MD2-MSCs. (C) Expression of ATP7A in WT- and MD-MSCs. ATP7A protein was not detected in MD1- and MD2-MSCs. (TIFF 936 kb) Additional file 7: Figure S6. Relative expression of *ATP7A* after siRNA transfection. The data are represented as the mean ± SE (*n* = 3); ***p* < 0.01. (TIFF 326 kb) Additional file 8: Figure S7. Osteogenesis and chondrogenesis from MSCs. (A) SMAD1 phosphorylation in WT- and MD-MSCs during osteogenesis. (B) Differentiation of WT- and MD-MSCs into chondrocytes. WT- and MD-MSCs differentiated into chondrocytes following the procedures described in the Materials and Methods section. Morphologies of chondrocyte spheroids were similar between WT- and MD-MSCs (upper panel). Scale bar = 500 μm. Additionally, MD-MSCs stained normally with Alcian blue solution (lower panel). Scale bar = 50 μm. (TIFF 1998 kb) Additional file 9: Figure S8. Activity of LOX and matrix collagen in WT- and MD-OBs. (A) LOX activity in WT- and MD-OBs. Fluorescence data showing the LOX activity of MD-OBs (see the Materials and Methods section) are expressed as values relative to those of WT-OBs. The data are presented as the mean ± SE (*n* = 3); **p* < 0.05, ***p* < 0.01. (B) Relative amount of matrix collagen in WT- and MD-OBs. The absorbance data obtained from a matrix collagen assay (see the Materials and Methods section) are expressed as values relative to that of WT-OBs. The data are presented as the mean ± SE (*n* = 3). (TIFF 813 kb)

## Abbreviations

ALP: Alkaline phosphatase
α-MEM: α-Minimum essential medium
BMP2: Bone morphogenetic protein 2
BSA: Bovine serum albumin
DAPI: 4′-6-diamidino-2-phenylindole
DMEM: Dulbecco’s modified Eagle’s medium
DW: Distilled water
EBs: Embryoid bodies
ECM: Extracellular matrix
ESC: Embryonic stem cell
FBS: Fetal bovine serum
FGF: Fibroblast growth factor
H&E: Hematoxylin and eosin
HRP: Horseradish peroxidase
ICP: Inductively coupled plasma
iPSC: Induced pluripotent stem cell
LOX: Lysyl oxidase
MD: Menkes disease
MMC: Mitomycin C
MSC: Mesenchymal stem cell
OB: Osteoblast
OHS: Occipital horn syndrome
PBS: Phosphate-buffered saline
PBST: Phosphate-buffered saline + 0.1 % Tween 20
Pi: Inorganic phosphate
PI: Propidium iodide
PPI: Pyrophosphate
RT-PCR: Real-time polymerase chain reaction
SB: SB431542
WT: Wild type
