# Supplementary figures and images for: Impaired osteogenesis in Menkes disease-derived induced pluripotent stem cells
Source: Stem Cell Res Ther. 2015 Sep 7;6(1):160. doi: 10.1186/s13287-015-0147-5 (PMC4562349; doi:10.1186/s13287-015-0147-5)

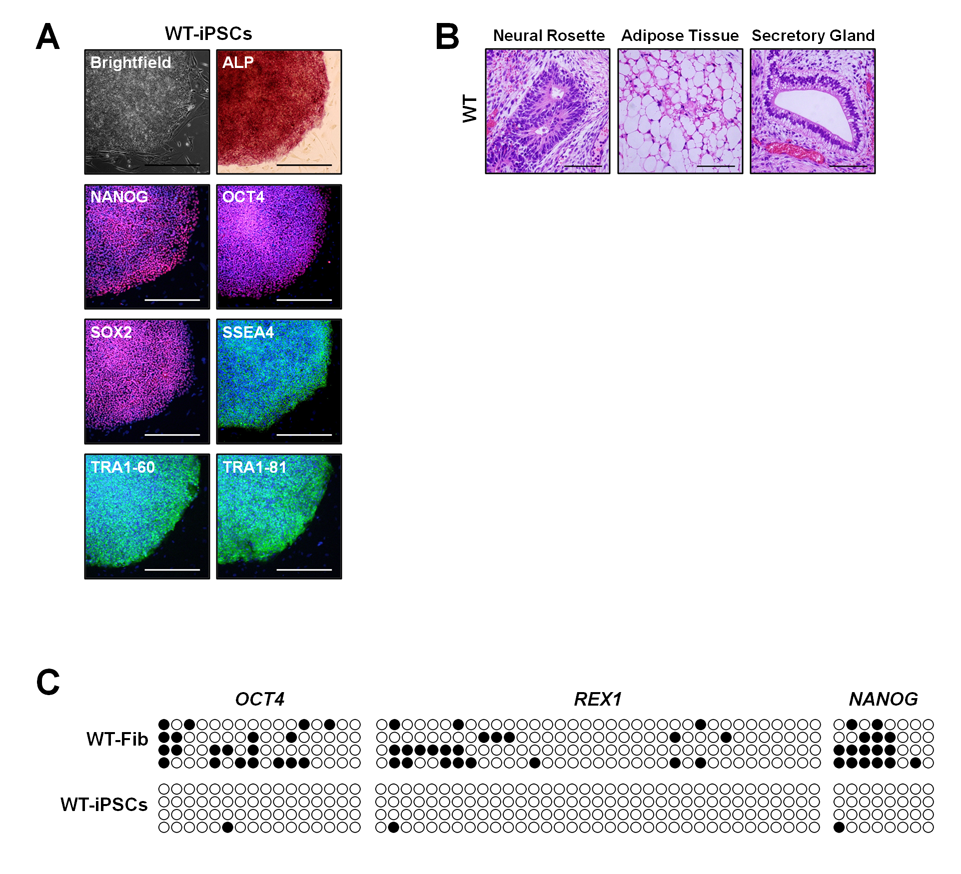

Supplement: Additional file 1: Figure S1. — Characterization of WT-iPSCs. (A) Expression of pluripotent markers in WT-iPSCs. (B) Teratoma formation of MD-iPSCs in immunodeficient mice. (C) Epigenetic reprogramming in WT-iPSCs. (TIFF 2486 kb) [file 13287_2015_147_MOESM1_ESM.tiff]

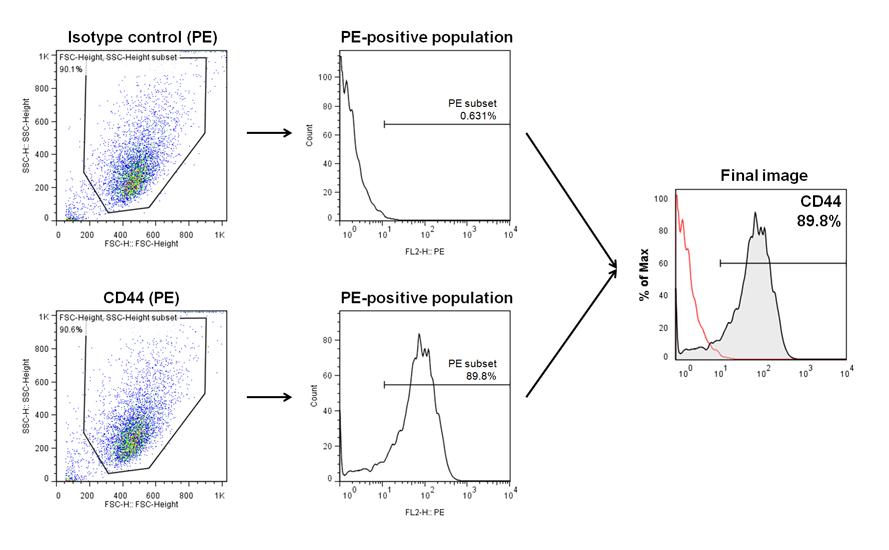

Supplement: Additional file 3: Figure S2. — Detailed gating strategy for each MSC surface antigens. Gating strategy for CD44 surface antigen shown as a representative. Live cells (gate A) were gated based on forward scatter and side scatter. Staining with isotype control (PE-conjugated) was used to exclude CD44-negative events. This gate was then applied to samples stained with anti-CD44 antibody (PE-conjugated) to identify CD44 positive events. In merged image, red line indicates isotype control and black tinted area indicates CD44 positive events. (TIFF 1455 kb) [file 13287_2015_147_MOESM3_ESM.tiff]

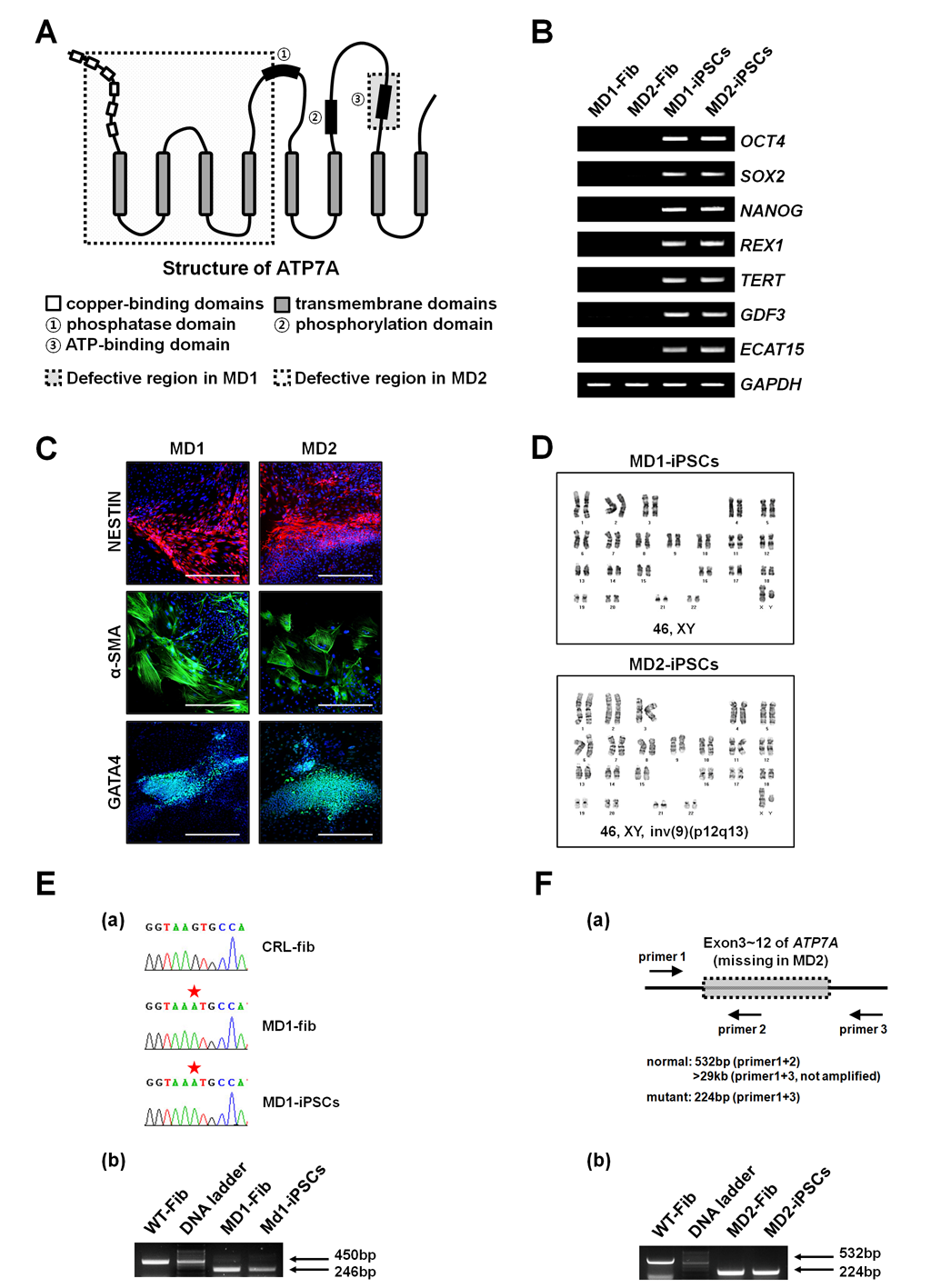

Supplement: Additional file 4: Figure S3. — Characterization of MD-iPSCs. (A) Schematic defective regions of ATP7A in MD1 and MD2 patients. The functional domains of ATP7A are depicted. ATP7A has six copper-binding domains, one phosphatase domain, one phosphorylation domain, one ATP-binding domain, and eight transmembrane domains. The defective regions of each patient are marked as boxes. (B) Transcriptional expression of pluripotent genes in MD-fibroblasts and MD-iPSCs. GAPDH was used as a control. (C) In vitro differentiation of MD-iPSCs. Immunostaining of NESTIN (ectoderm, red), α-SMA (mesoderm, green), and GATA4 (endoderm, green) was performed 7 days after spontaneous EB differentiation. DAPI showed nuclear counterstaining (blue). Scale bar = 500 μm. (D) Karyotypes of MD1- and MD2-iPSCs. (E) Genetic mutations in MD1-fibroblasts and MD1-iPSCs. A single-base substitution was confirmed in MD1-fibroblasts and MD1-iPSCs (Figure E-a). The resultant PCR products were different in MD1-fibroblasts and MD1-iPSCs (Figure E-b). The size difference (450 bp in WT and 246 bp in MD1) generated by exon skipping was analyzed by gel electrophoresis. (F) Mutations in MD2-fibroblasts and MD2-iPSCs. Strategy for duplex PCR was explained as an illustration (Figure F-a). Size differences (532 bp in WT and 224 bp in MD2) generated by a large genomic deletion between WT- and MD2-fibroblasts were detected (Figure F-b). The primers used in this study are listed in Additional file 1 (Table S2). (TIFF 4321 kb) [file 13287_2015_147_MOESM4_ESM.tiff]

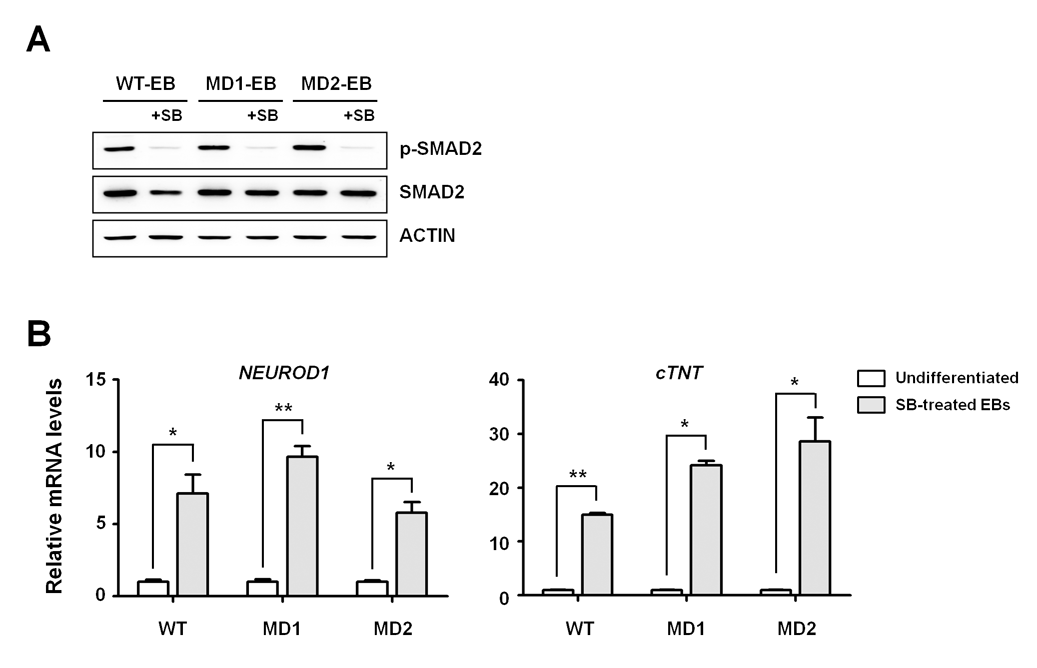

Supplement: Additional file 5: Figure S4. — Characterization of EBs during MSC differentiation. (A) SMAD2 phosphorylation in WT- and MD-EBs during MSC differentiation. SB treatment suppressed p-SMAD2 in WT- and MD-EBs. (B) Relative expression of neuro-ectoderm and cardiac mesoderm marker genes (NEUROD1 and cTNT, respectively) in WT- and MD-EBs. The data are presented as the mean ± SE (n = 3); *p < 0.05, **p < 0.01. (TIFF 2052 kb) [file 13287_2015_147_MOESM5_ESM.tiff]

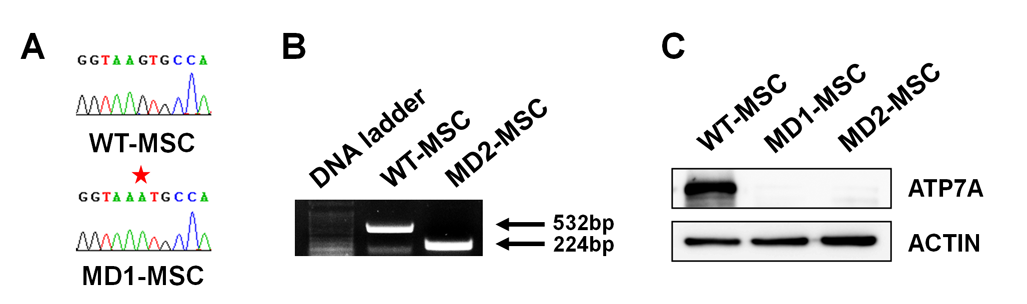

Supplement: Additional file 6: Figure S5. — Confirmation of MD-MSCs. (A) Genetic mutation of the ATP7A gene in MD1-MSCs. A single-base substitution was confirmed again in MD1-MSCs. (B) Genetic mutation of the ATP7A gene in MD2-MSCs. Size differences in the PCR product were observed in MD2-MSCs. (C) Expression of ATP7A in WT- and MD-MSCs. ATP7A protein was not detected in MD1- and MD2-MSCs. (TIFF 936 kb) [file 13287_2015_147_MOESM6_ESM.tiff]

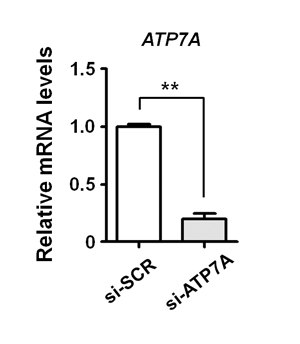

Supplement: Additional file 7: Figure S6. — Relative expression of ATP7A after siRNA transfection. The data are represented as the mean ± SE (n = 3); **p < 0.01. (TIFF 326 kb) [file 13287_2015_147_MOESM7_ESM.tiff]

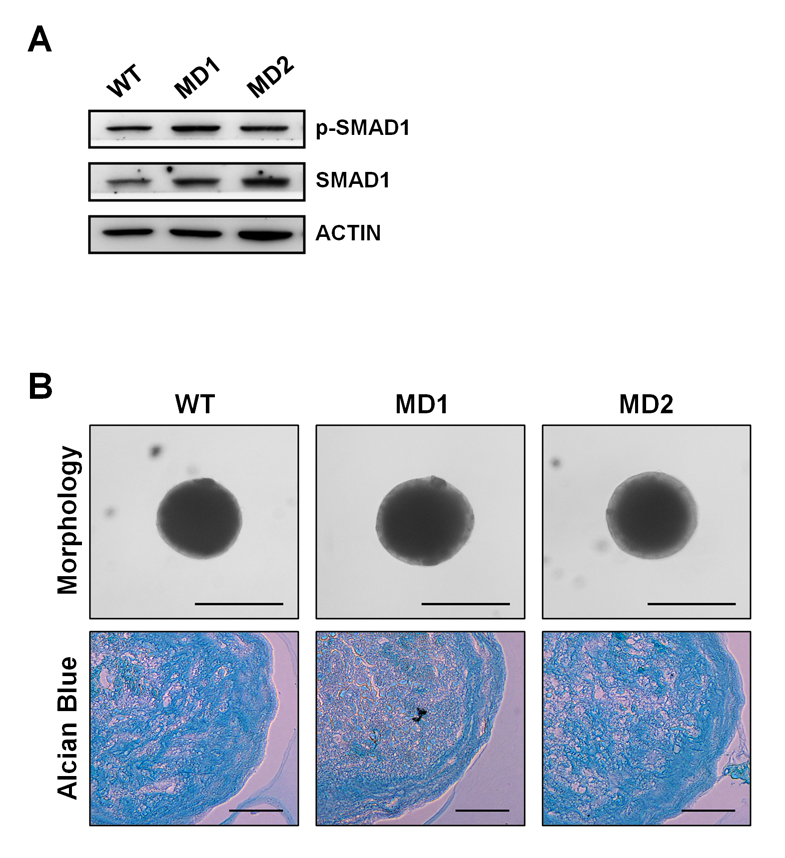

Supplement: Additional file 8: Figure S7. — Osteogenesis and chondrogenesis from MSCs. (A) SMAD1 phosphorylation in WT- and MD-MSCs during osteogenesis. (B) Differentiation of WT- and MD-MSCs into chondrocytes. WT- and MD-MSCs differentiated into chondrocytes following the procedures described in the Materials and Methods section. Morphologies of chondrocyte spheroids were similar between WT- and MD-MSCs (upper panel). Scale bar = 500 μm. Additionally, MD-MSCs stained normally with Alcian blue solution (lower panel). Scale bar = 50 μm. (TIFF 1998 kb) [file 13287_2015_147_MOESM8_ESM.tiff]

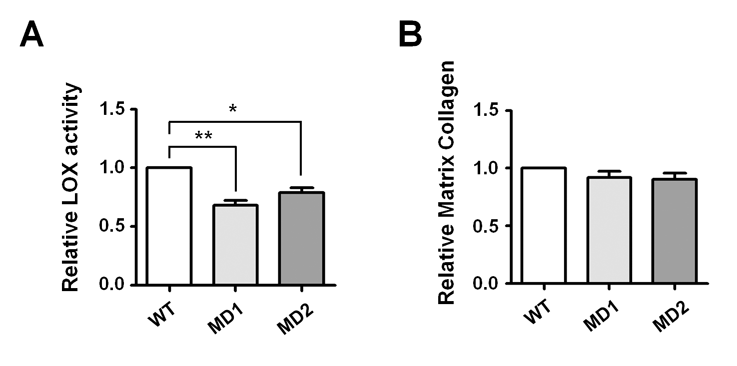

Supplement: Additional file 9: Figure S8. — Activity of LOX and matrix collagen in WT- and MD-OBs. (A) LOX activity in WT- and MD-OBs. Fluorescence data showing the LOX activity of MD-OBs (see the Materials and Methods section) are expressed as values relative to those of WT-OBs. The data are presented as the mean ± SE (n = 3); *p < 0.05, **p < 0.01. (B) Relative amount of matrix collagen in WT- and MD-OBs. The absorbance data obtained from a matrix collagen assay (see the Materials and Methods section) are expressed as values relative to that of WT-OBs. The data are presented as the mean ± SE (n = 3). (TIFF 813 kb) [file 13287_2015_147_MOESM9_ESM.tiff]
