# Supplementary material for: Impaired osteogenesis in Menkes disease-derived induced pluripotent stem cells
Source: Stem Cell Res Ther. 2015 Sep 7;6(1):160. doi: 10.1186/s13287-015-0147-5 (PMC4562349; doi:10.1186/s13287-015-0147-5)
Supplement: Additional file 2: Table S1. — Primers used in RT-PCR analysis. Table S2 Primers used in ATP7A genotyping of MD-derived cells. Table S3 Primers used in methylation analysis. Table S4 Copper concentration in WT- and MD-MSCs. (DOCX 19 kb) [file 13287_2015_147_MOESM2_ESM.docx]

**Table S1 Primers used in RT-PCR analysis**

| GENE  (Genbank ID) | Forward Sequence | Reverse Sequence |
| --- | --- | --- |
| *OCT4*  (NM_002701.5) | GTACTCCTCGGTCCCTTTCC | CAAAAACCCTGGCACAAACT |
| *SOX2*  (NM_003106.3) | CCCAGCAGACTTCACATGT | CCTCCCATTTCCCTCGTTTT |
| *NANOG*  (NM_024865.2) | CAGCCCTGATTCTTCCACCAGTCCC | GGAAGGTTCCCAGTCGGGTTCACC |
| *REX1*  (NM_174900.3) | CAGATCCTAAACAGCTCGCAGAAT | GCGTACGCAAATTAAAGTCCAGA |
| *TERT*  (NM_198253.2) | TGTGCACCAACATCTACAAG | GCGTTCTTGGCTTTCAGGAT |
| *GDF3*  (NM_020634.1) | AAATGTTTGTGTTGCGGTCA | TCTGGCACAGGTGTCTTCAG |
| *ECAT15*  (NM_018189.3) | GGAGCCGCCTGCCCTGGAAAATTC | TTTTTCCTGATATTCTATTCCCAT |
| *GAPDH*  (NM_002046.4) | GAAGGTGAAGGTCGGAGTC | GAAGATGGTGATGGGATTTC |
| *OCT4*  Transgene | GTACTCCTCGGTCCCTTTCC | CCCTTTTTCTGGAGACTAAATAAA |
| *SOX2*  Transgene | CATGTCCCAGCACTACCAGA | CCCTTTTTCTGGAGACTAAATAAA |
| *cMYC*  Transgene | AAGAGGACTTGTTGCGGAAA | CCCTTTTTCTGGAGACTAAATAAA |
| *KLF4*  Transgene | GAACTGACCAGGCACTACCG | CCCTTTTTCTGGAGACTAAATAAA |
| *RUNX2*  (NM_001278478.1) | TAGGCGCATTTCAGATGATG | GACTGGCGGGGTGTAAGTAA |
| *OPN*  (NM_001040058.1) | ACAGCCAGGACTCCATTGAC | ACACTATCACCTCGGCCATC |
| *OCN*  (NM_199173.4) | GGCAGCGAGGTAGTGAAGAG | AGCAGAGCGACACCCTAGAC |
| *NEUROD1*  (NM_002500.4) | GTTCTCAGGACGAGGAGCAC | GTCTCTTGGGCTTTTGATCG |
| *cTNT*  (NM_000364.3) | TGGAGGAGTCCAAACCAAAG | ATTCAGGTCCTTCTCCATGC |

**Table S2 Primers used in ATP7A genotyping of MD-derived cells.**

| Gene | SEQUENCE | DESCRIPTION |
| --- | --- | --- |
| ATP7A  (NM000052.6) | F: GCTTGTTATTGCTCAGTTATGT R: TATGACATTATCATTGACCACA | For detecting splice-site mutation in gDNA  (JBM mutation) |
|  | F: AGAGAAAAGGTCGGACTGCTG R: TCCGAATCCTCTTGACTGTCTC | For detecting exon20 skipping in cDNA  (JBM mutation) |
|  | F: GAGAATCCCAGTTCCTGACG R1: GACTGTTGGGAAGGCATGAT R2: TAAGCCTGCCGGTTTATGAG | For detecting exon3-12del (Duplex PCR)  (KGB mutation) |

**Table S3 Primers used in methylation analysis.**

| Gene | SEQUENCE | GENOMIC REGION (TSS=+1) |
| --- | --- | --- |
| *NANOG*  (NM_024865.2) | F: TGGTTAGGTTGGTTTTAAATTTTTG R: AACCCACCCTTATAAATTCTCAATTA | -387 ~ -52 |
| *OCT4*  *(NM_002701.5)* | F1: ATAAAGTGAGATTTTGTTTTAAAAA R1: AACATAAAAAAATCCCCCACAC F2: GGGATTTGTATTGAGGTTTTGG R2: CCCACACCTCAAAACCTAAC | -204 ~ +229 |
| *REX1*  (NM_174900.3) | F: GGTTTAAAAGGGTAAATGTGATTATATTTA R: CAAACTACAACCACCCATCAAC | -428 ~ -68 |

**Table S4** **Copper concentration in WT- and MD-MSCs.**

|  | WT-MSCs | MD1-MSCs | MD2-MSCs |
| --- | --- | --- | --- |
| Copper concentration  (ng of copper/mg of protein) | 47.5 ± 10.5 | 69.7 ± 1.0 | 63.8 ± 2.0 |
